# The Eµ-hnRNP K Murine Model of Lymphoma: Novel Insights into the Role of hnRNP K in B-Cell Malignancies

**DOI:** 10.3389/fimmu.2021.634584

**Published:** 2021-04-12

**Authors:** Prerna Malaney, María Velasco-Estevez, Pedro Aguilar-Garrido, Marisa J. L. Aitken, Lauren E. Chan, Xiaorui Zhang, Sean M. Post, Miguel Gallardo

**Affiliations:** ^1^ Department of Leukemia, MD Anderson Cancer Center, Houston, TX, United States; ^2^ H12O–CNIO Haematological Malignancies Clinical Research Unit, CNIO, Madrid, Spain

**Keywords:** B-cell malignancies, lymphoma, diffuse large B cell lymphoma, mouse model, hnRNP K, *Eµ-Hnrnpk*, RNA-binding protein, *MYC*

## Abstract

B-cell lymphomas are one of the most biologically and molecularly heterogeneous group of malignancies. The inherent complexity of this cancer subtype necessitates the development of appropriate animal model systems to characterize the disease with the ultimate objective of identifying effective therapies. In this article, we discuss a new driver of B-cell lymphomas – hnRNP K (heterogenous nuclear ribonucleoprotein K)—an RNA-binding protein. We introduce the *Eµ-Hnrnpk* mouse model, a murine model characterized by hnRNP K overexpression in B cells, which develops B-cell lymphomas with high penetrance. Molecular analysis of the disease developed in this model reveals an upregulation of the *c-Myc* oncogene *via* post-transcriptional and translational mechanisms underscoring the impact of non-genomic *MYC* activation in B-cell lymphomas. Finally, the transplantability of the disease developed in *Eµ-Hnrnpk* mice makes it a valuable pre-clinical platform for the assessment of novel therapeutics.

## Introduction to B-Cell Malignancies

B-cell neoplasms comprise a heterogeneous cohort of hematological malignancies originating from B cells. They are classified, according to WHO criteria, into precursor B-cell neoplasms (B-lymphoblastic leukemia) and mature B-cell neoplasms (1). Mature B-cell neoplasms encompass a plethora of malignancies, such as leukemias (chronic lymphocytic leukemia, small lymphocytic lymphoma), plasma cell malignancies (e.g. myeloma), and lymphomas. Specifically, lymphomas are classified as Hodgkin’s and non-Hodgkin’s lymphomas, which account for 10% and 90% of cases, respectively (2). Nearly half of all non-Hodgkin’s lymphomas are diffuse large B-cell lymphomas (DLBCL), making them the most commonly diagnosed lymphoma sub-type. Other non-Hodgkin’s lymphomas include follicular lymphomas, marginal zone lymphoma, mantle cell lymphoma and Burkitt’s lymphoma (3).

B cells are part of the adaptive immune system and play a vital role in the production of antigen-specific immunoglobulins (antibodies) in response to invasive pathogens. In order to do so, B cells must maintain an extensive array of antigen receptors. B cells must also demonstrate high variability in antigen recognition sites in order to protect organisms against a plethora of pathogens. To achieve this, B cells employ a complex mechanism called VDJ recombination, which enables the generation of an almost unlimited repertoire of antigenic B-cell receptors. The VDJ recombination process involves the formation and repair of several highly regulated DNA breaks. Aberrations in this intricate process along with spontaneous mutations and breaks in the DNA lead to alterations that in turn promote the malignant transformation of B cells (4, 5).

Aberrant VDJ recombination often results in chromosomal translocations. A clear example of this is Burkitt’s lymphoma wherein a chromosomal translocation results in constitutive activation of the *MYC* oncogene (6–8). Other examples include the t(14;18) translocation in follicular lymphoma, resulting in constitutive expression of *BCL2* (4) and the t(11;14) translocation involving the cyclin D1 gene in mantle-cell lymphoma (9, 10). In addition to translocations, somatic mutations in various genes have also been implicated in the pathogenesis of other non-Hodgkin’s lymphomas (11). Beyond mutations, overexpression of oncogenes and loss of tumor suppressor genes have also been identified as driver events for lymphomagenesis (12–14). Well-defined genetic basis of disease has helped establish several genetically defined transgenic models of B-cell malignancies, as discussed below.

Microscopic analysis of lymphomas show that tumors contain not only cancerous cells, but also host immune cells, stromal cells, blood vessels and the extracellular matrix, comprising the *tumor microenvironment* (15). Tumor cells create this microenvironment by first homing to sites that promote or help their growth, and later recruiting support cells and/or causing cells in their microenvironment to differentiate to their benefit (3). In recent years, it has become clear that the tumor microenvironment is a key player in the onset and progression of cancer, as well as therapy resistance, underscoring the need for better understanding in this area. Investigating B-cell lymphomas using *in vitro* systems is an important initial approach, but its predictive value is limited as it leaves out other factors such as the host immune system and the tumor microenvironment. Therefore, the use of more complex tools such as genetically engineered mouse models are needed to more fully study B-cell malignancies.

### Need for the Development of New Murine Models for B-Cell Malignancies

Murine models of hematological malignancies can be classified as genetically modified models or patient-derived xenograft (PDX) models. The former can be used to investigate the onset, progression and therapeutic approaches for leukemia and lymphomas, while the latter is a valuable platform for drug testing and pre-clinical research.

PDX models are ideal for preclinical studies, wherein a heterogeneous population of tumor cells extracted from a patient is implanted into an immunocompromised mouse, thereby facilitating studies that assess the response or resistance of the disease to existing and novel therapeutic agents (16). Despite the caveat of lacking the tumor-immune cell component, B-cell lymphoma PDX mouse models can show biological, histopathological and clinical features of the original patient tumor, making it an ideal platform for preclinical studies (17). The use of humanized mice for transplantation is gaining popularity and allows for the assessment of immunotherapies as well as the study of the interactions between the tumor and the immune system (18). However, PDX models cannot be used to investigate the onset and progression of tumors. They are also not amenable for the identification of novel oncogenes or tumor suppressor genes, making transgenic and knockout mouse models the preferred platform for this aim. While PDX models enable the assessment of developed disease, transgenic animal models can be used to investigate the origin and onset of disease. Moreover, PDX models carry the diversity of the patients they are derived from and are therefore diverse in their pathology and drug responses, complicating interpretation of data. Transgenic models, however, have a common background allowing for a replicable and controlled phenotype for research. Taken together, the research hypotheses and objectives must be taken into consideration while selecting an appropriate animal model.

There are several well-defined genetic alterations observed in human patients that give rise to B-cell lymphomas due to uncontrolled B-cell proliferation and/or maturation. Some of these genetic alterations have already been recreated in transgenic mice to study the spontaneous onset and progression of lymphomas and leukemia-like phenotypes. Notable models include a *BCL6* knock-in which results in a DLBCL-like phenotype (19), *VavP-Bcl2* mice, mimicking the t(14; 18) chromosomal translocation, which develop follicular lymphomas, a targeted deletion of *Trp53* – *CD21-Tp53lox* – that develops non-germinal center B-cell lymphomas, and *Eµ-Tcl1* mice which develop aggressive chronic lymphocytic leukemia (20–22).

However, the most recurrent and extensively investigated genetic alterations are translocations of the *MYC* oncogene. In the 1980s, *MYC* was identified as the first proto-oncogene in B-cell lymphomas. Transgenic mouse models with translocations in the *Myc* gene were first introduced in 1985 (23). Of these models, the most extensively used is the *Eµ-Myc* model. This mouse obtained by the translocation of *Myc* to the Ig Heavy chain (IgH) locus, causing overexpression of c-Myc and abnormal B-cell proliferation (24). Similar to humans, *Eµ-Myc* mice develop B-cell lymphoma-like malignancies with a >90% penetrance and variable onset. These *Eµ-Myc* mice exhibit either a DLBCL phenotype or a Burkitt’s lymphoma phenotype depending on the time of development (25, 26). Another model of c-Myc driven Burkitt’s lymphoma is one driven solely by the 3’ regulatory region of the IgH locus rather than the *Eµ* enhancer. This model results in the development of B-cell malignancies with a mature B-cell phenotype in contrast to the *Eµ* model wherein a pro-B phenotype is predominant (27, 28). However, multiple mouse models with other *Myc* translocations develop different B-cell malignancies, such as *Vκ-Myc* mice for myeloma (29), *λ-Myc* for Burkitt’s lymphoma (30), iMycEμ for endemic Burkitt’s lymphoma (31), and iMycCμ mice for sporadic and immunodeficiency-associated Burkitt’s lymphoma (32), demonstrating the critical role of c-Myc in B cell biology.

## New Driver of B-Cell Lymphomas: hnRNP K

Altered genes have historically been designated as either tumor suppressors or oncogenes. However, recent studies have shown that some genes can have dual oncogenic and tumor-suppressive functions in different contexts (33). One such example of a dual regulator is heterogeneous nuclear ribonucleoprotein K (*HNRNPK*) (34).

hnRNP K is an ss-DNA and RNA-binding protein that regulates a myriad of cellular processes *via* transcriptional, posttranscriptional and translational mechanisms. It contains three K homology (KH) domains – responsible for nucleic acid binding – one K-protein-interactive domain (KI), and one nuclear-cytoplasmic shuttling domain (KNS) (35, 36). Due to hnRNP K’s pleiotropic nature, both its over- and under-expression can be pathogenic (37–41), likely by deregulating the transcription and/or translation of multiple cellular oncogenes or tumor suppressors. For instance, there is a clinical correlation between high levels of hnRNP K and the onset and treatment resistance of various tumors types in patients, such as lung (42), breast (43), rectal adenocarcinoma (44), and melanoma (45). In the context of hematological malignancies, ~2% of acute myeloid leukemia patients have a 9q21.32 deletion that encompasses the *HNRNPK* gene, resulting in loss of one copy of *HNRNPK*. Consequently, haploinsufficiency of *HNRNPK* was shown to be pathogenic in mice, as an *Hnrnpk^+/-^* mouse model showed a myeloproliferative phenotype and reduced survival. In addition, *Hnrnpk* haploinsufficiency also triggered B-cell lymphoma phenotypes in 30% of the animals. This role of hnRNP K as a tumor suppressor is partially due to its regulation of the p53/p21 pathway (40, 46). However, it is of note that hnRNP K acts not only as a tumor suppressor, but also an oncogene. It has been shown that hnRNP K promotes the expression of classical oncogenes such *c-Myc*, resulting in the development of B-cell malignancies (39, 45), as well as c-Src (47) and eIF4E (38). As an ss-DNA and RNA-binding protein, hnRNP K can regulate both the transcription and translation of certain genes, as it does in the case of *MYC*. It has been observed that hnRNP K binds to the CT-rich regions of the *MYC* promoter, enhancing transcription of this gene (48–50). Additionally, hnRNP K positively regulates translation of *MYC* mRNA by binding to the IRES (Internal Ribosome Entry Site) sequence, aiding its entry to the ribosome through a cap-independent mechanism (37, 51). Control of *MYC* translation *via* this mechanism through hnRNP K is significant in the context of tumorigenesis, as it has been observed that even a single CT mutation in the IRES sequence – present in 42% of myeloma multiple patients – could enhance the binding of hnRNP K to this sequence and promote an increased aberrant expression of c-Myc (52). In fact, hnRNP K has been described as an (IRES)-trans-acting factors (ITAF) and can potentially regulating cap-independent translation of several oncogenes resulting in the systemic activation of an oncogenic program (53, 54).

Given the role of hnRNP K in regulating the expression levels of oncogenic molecules, elevated hnRNP K levels are observed in several solid and hematological malignancies (42, 44, 45). Specific to B-cell lymphomas, as we have previously described in Gallardo et al., hnRNP K is overexpressed in human patients with diffuse large B-cell lymphoma and is associated with poor clinical outcomes and non-responsiveness to chemotherapy (39). Overexpression of hnRNP K is also observed in Burkitt’s lymphoma where sumoylated hnRNP K is elevated, which regulates the expression of c-Myc at the translational level (55). Taken together, a dual role of hnRNP K has been observed in the formation of hematological malignancies, wherein an under-expression of the protein leads to a deficit in its role inducing the expression of tumor suppressors p53/p21, thus leading to a myeloproliferative phenotype and lymphoma in rodents (40), while on the other hand, an increment in the levels of hnRNP K has been related to higher levels of c-Myc and the formation of lymphomas (39, 55).

Critically, with respect to B-cell malignancies, we observe an oncogenic function for hnRNP K. Our data showed that hnRNP K is overexpressed in DLBCL patients and higher expression of hnRNP K correlates with poor clinical outcomes and lack of response to chemotherapy in these patients. Interestingly, we observed that hnRNP K is overexpressed in patients who do not harbor any *MYC* genomic alterations. The regulation of *MYC* by hnRNP K occurs at the post-transcriptional and translational level, indicating that hnRNP K over-expression represents a key non-genomic mechanism of *MYC* regulation in B-cell lymphomas (37).

The role of hnRNP K is especially critical given the increasing clinical relevance of RNA-binding proteins (RBPs) and splicing factors in hematological malignancies. Mutations and aberrant expression of RBPs is increasingly being observed in leukemia, myelodysplastic disease, and lymphomas (56–60). Moreover, taking into consideration the critical role of c-Myc not only in B-cell biology and B-cell malignancies, but also in other heme malignancies, hnRNP K-mediated regulation of c-Myc warrants extensive study. The B-cell specific murine model of hnRNP K, *Eµ-Hnrnpk*, described below, is therefore crucial for unraveling the complex and nuanced role of this protein in B-cell malignancies.

## *Eµ-Hnrnpk* Murine Model as a Platform to Study B-Cell Malignancies

We previously generated the *Eµ-Hnrnpk* mouse model by placing the *Hnrnpk* cDNA downstream of the extensively characterized immunoglobulin heavy-chain (*Igh*) enhancer Eµ. The resulting mice specifically overexpress hnRNP K in B cells, have reduced survival, and develop B-cell malignancies (39). The *Eµ-Hnrnpk* model has several utilities and applications. First, the model helps establish the RNA-binding protein, hnRNP K, as a *bona fide* oncogene. To the best of our knowledge, this is the first published transgenic mouse model that demonstrates the oncogenicity of hnRNP K over-expression. Second, the model provides an *in-vivo* system to assay the role of hnRNP K in B-cell biology and malignancy. Third, the mice provide a pre-clinical platform to test existing and experimental therapeutics.

### Phenotype of the *Eµ-Hnrnpk* Mouse Model

The *Eµ-Hnrnpk* mice develop B-cell malignancies with a high latency. Roughly 70% of mice survived the first year but only 20% survived until the end of the second year. This accelerated mortality between years one and two may be due to yet-unknown secondary genomic aberrations or epigenetic changes warranting further in-depth analyses of this disease model. In contrast with the slow generation of the phenotype, the disease penetrance is incredibly high – almost 100% of the mice developed some type of B-cell malignancy.

Gross analyses revealed that *Eµ-Hnrnpk* mice have marked hepatosplenomegaly, a 12-fold increase in spleen weight, and a 3.5-fold increase in the hepatic weight. The splenomegaly is accompanied by loss of splenic architecture, expansion of B-cell lineages as evidenced by increased PAX5 staining, and the presence of highly proliferative Ki67+ cells. In addition to their proliferative nature, the malignant cells have invasive properties as well. Livers of tumor-burdened mice showed extensive B-cell (B220+) infiltration and minimal T-cell (CD3+) infiltration.

The malignancy of *Eµ-Hnrnpk* cells was confirmed through transplantation assays. Immunodeficient mice injected with cells from *Eµ-Hnrnpk* mice bearing disease recapitulate the same phenotypes (reduced survival, B cell proliferation), confirming the cell-autonomous nature of *Eµ-Hnrnpk* cells.

The preponderance of large malignant cells observed in hematopoietic tissues of the *Eµ-Hnrnpk* mice and clinical data from DLBCL patients pinpoint the role of hnRNP K overexpression in this disease. However, a small proportion (2-5%) of the *Eµ-Hnrnpk* mice developed other types of B-cell malignancies, not described herein, consistent with plasma cell-like malignancy. This observation falls in line with some late-arising tumors seen in *Eµ-Myc* mice that tend to develop lymphomas derived from plasma cells or with plasma cell differentiation (25).

### Molecular Mechanism of hnRNP K-Mediated Lymphomagenesis

**Figure 1:**
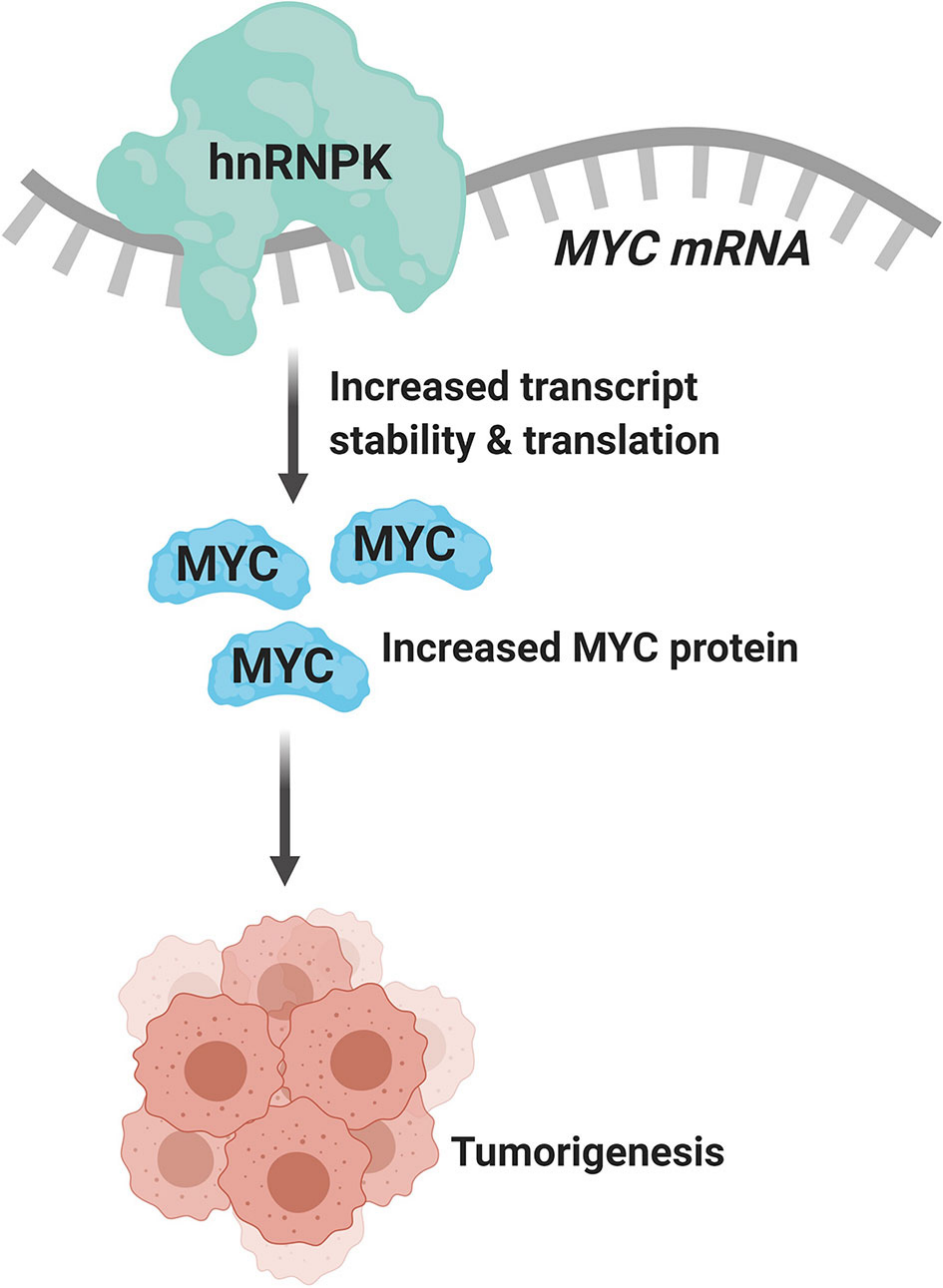
hnRNP K binds to and regulates the stability and translation of the *MYC* transcript (created with BioRender.com).

**Figure 2:**
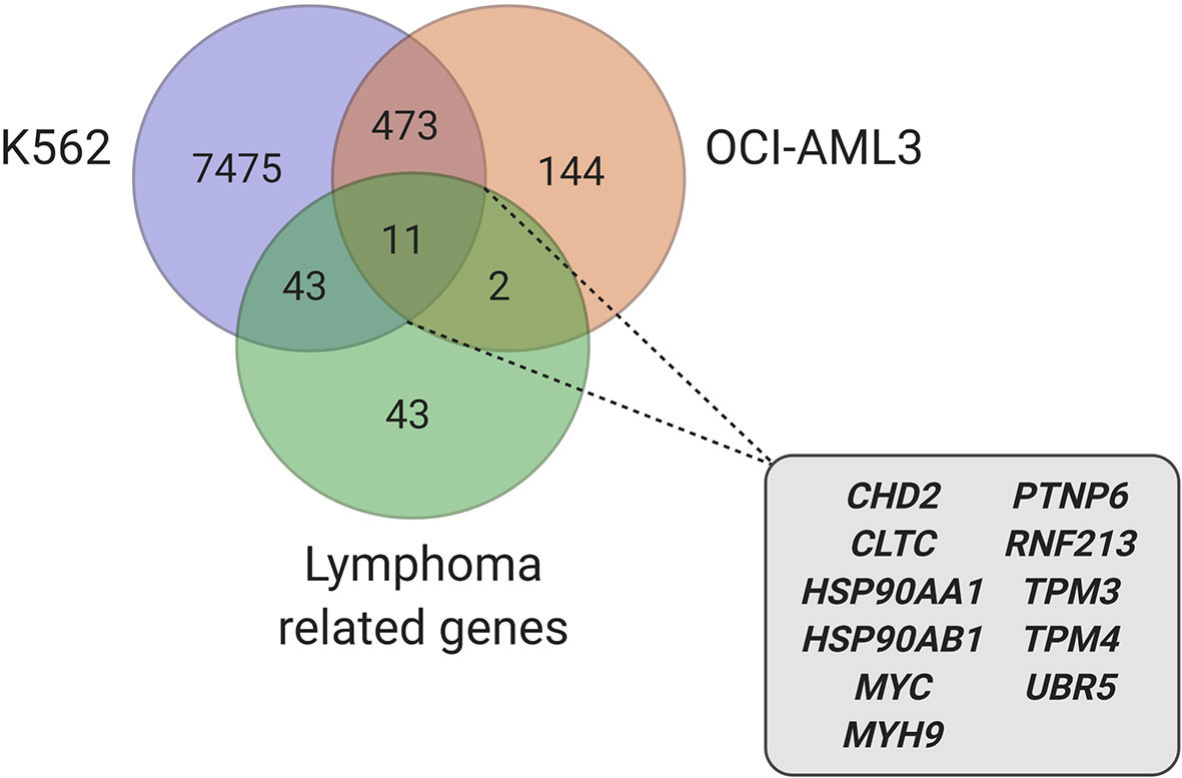
Candidate genes, involved in lymphoma, identified in hnRNP K RIP-Seq datasets (39, 64) (created with BioRender.com).

Due to the plurality of hnRNP K’s cellular roles, the molecular mechanism of hnRNP K-mediated lymphomagenesis is likely to be multifaceted and complex. However, hnRNP K has been previously demonstrated to bind to C-rich regions in DNA and RNA (38, 61). Data obtained from RIP-Seq (RNA immunoprecipitation followed by sequencing) and its formaldehyde-fixed molecular cousin (fRIP-seq) (62) experiments, previously published in Gallardo et al., revealed that one of the top targets of hnRNP K is the *MYC* transcript, and that this interaction enhances its stability and translation (39). We confirmed a direct interaction between hnRNP K protein and the *MYC* transcript using fluorescence anisotropy assays. Actinomycin-chase experiments revealed an increased stability of the *MYC* transcript associated with hnRNP K overexpression. Finally, polysome profiling assays revealed the essential role of hnRNP K in regulating *MYC* translation and that knockdown of hnRNP K adversely affected the loading of the *MYC* transcript onto monosomes. Taken together, our biochemical and molecular assays establish a physical and functional link between hnRNP K and *MYC* (**Figure 1**). This finding is borne out in the *in vivo* model: lymphomas derived from *Eµ-Hnrnpk* mice show elevated c-Myc levels. These findings are particularly relevant when coupled with the observation that hnRNP K expression levels are elevated in DLBCL patients without *MYC* genomic alterations, suggesting that hnRNP K can drive c-Myc signaling in the absence of *MYC* mutations. Therefore, hnRNP K overexpression represents an alternate mechanism of c-Myc pathway activation in B-cell malignancies. Interestingly, *Smurf2* ablation in mice also results in the development of B-cell tumors due to transcriptional upregulation of the c-Myc protein representing yet another non-genomic mechanism of aberrant c-Myc expression in tumors (63). Considering the fact that hnRNP K binds to a multitude of transcripts, it would be reasonable to assume that it causes lymphomagenesis *via* several mechanisms and not just *via* regulation of c-Myc. An overlap of RIP-Seq data from two hematological cell lines (K562 and OCI-AML3) with a set of genes implicated in lymphomas reveal several interesting candidates for further study (**Figure 2**) (19, 64).

Beyond its RNA-binding functions, hnRNP K also interacts with a host of proteins *via* its KI domain. Therefore, it is entirely plausible that the protein interactome of hnRNP K contributes to its oncogenicity. Immunoprecipitation of hnRNP K followed by mass spectrometry, as described in Gallardo et al., revealed that hnRNP K associates with a host of ribosomal and RNA processing proteins (39). Given the preponderance of ribosomal subunits in the hnRNP K interactome, the impact that hnRNP K has on global translational profiles and its relationship to p53 allude to the possibility that aberrations in hnRNP K are likely to contribute to a nucleolar stress response (NSR). In fact, IP/MS experiments reveal a physical interaction between hnRNP K and the NSR sensor Nucleolin (NCL). The impact of hnRNP K on ribosome biogenesis and nucleolar stress pathways and their relevance to disease is currently under investigation in our laboratories.

In conclusion, the *Eµ-Hnrnpk* mice demonstrate a level of biological heterogeneity that is also seen in human disease. Consequently, the model represents a valuable tool for the assessment of existing and novel drugs and therapies. The transplantability of malignant cells from this model and the aggressive nature of the transplanted disease allow for a rapid evaluation of therapeutic modalities. Although current transplantation studies with the *Eµ-Hnrnpk* are limited to immunodeficient mice, future research with syngeneic models may be of value, particularly to test immunotherapeutic agents.

## Discussion

The generation, maturation, and genetic reprogramming of B-cell lymphocytes represent some of the most complex biological processes outside of development. The maturation process occurs in a bevy of host hematologic tissues. Germinal centers (GCs) – specific structures in secondary lymphoid organs, such as lymph nodes and the spleen – are where the generation and selection of B cells occurs. GCs are typically divided into the dark and the light zone. In the dark zone, B cells undergo rapid and mutative cell division called immunoglobulin somatic hypermutation (SHM), while in the light zone, B cells are selected based on antigen affinity. The whole process of GC initiation, dark zone formation, and the passage of B cells between the dark and light zones to final differentiation is a concatenated process involving the participation of a plethora of proteins such as BCL6, BCL2, TCL1, PAX5, IRF4, NFKB, MLL2 and c-Myc (65). All of these molecules are differentially expressed based on areas within the GC and/or stage of B-cell differentiation and are intricately balanced *via* multiple feedback loops. The inherent process of B-cell maturation and function relies on the inaccuracy of targeted DNA breaks and subsequent repair during VDJ recombination, SHM, and class-switch recombination (CSR). The rapid mutative divisions in B cells therefore put their genomic integrity in jeopardy. When an error occurs, the two most common genomic sequelae are chromosomal translocations and aberrant SHMs (ASHMs) (66). These alterations are due to mistakes in recombination activating gene (RAG) mediated VDJ recombination process, mistakes in the activation-induced cytidine deaminase (AID) dependent-CSR process, or translocations involving IGH (66). The result of these aberrancies is commonly the juxtaposition of strong promoters/enhancers (e.g. IGH) in front of oncogenes related with B-cell biology. The dysregulation of these oncogenes drives B-cell hematological malignancies such as follicular lymphoma (*BCL2* translocation, MLL2 inactivation) (11, 67) DLBCL (e.g. *BCL6*, *BCL2* and *MYC* translocation) (8, 68, 69) or Burkitt’s lymphoma (*MYC* translocation) (70, 71).

In the last 40 years, researchers have tried to reproduce the same genetic alterations and translocations found in human B-cell malignancies in animal models in order to develop tools to study the pathophysiology of the disease and develop pre-clinical therapeutic platforms. However, there are some challenges involved in the creation of these models. The vast majority of models of B-cell disease – such as *Eµ-Myc*, *VavP-Bcl2* or *Iµ-Bcl6* – are not “clean” models, i.e. they do not perfectly recapitulate human disease or often result in the development of multiple phenotypes due to two main reasons. First, “timing” and “location” in B-cell development plays a pivotal role in its biology, and heavily impact the development of B-cell malignancies. Thus, expression of the oncogenes in a more mature or immature B-cell subtype could result in completely different phenotypes than those observed in human disease. Consequently, the use of universal B-cell promoters that are common to multiple B-cell subtypes will often result in mixed phenotypes (25). Second, most drivers of B-cell malignancies are master regulators of a slew of different biological processes. Therefore, dysregulation of these molecules inhibits DNA damage response, increases genomic instability, or promotes other biological phenomena that enhance the accumulation of genomic aberrations in lymphoma cells. These genetic alterations will likely be different in each individual mouse, and its relationship with the driver alteration could explain the diversity of phenotypes. As an example, Lefebure et al. studied the genetic landscape of *Eµ-Myc* mice and observed multiple single nucleotide variants, deletions, or indels in *Bcor*, *Trp53*, *Cdkn2a, Kras* or *Nras*, suggesting that genomic instability driven by the genetic modification i.e., *Myc*, and the collaboration of other oncogenes and tumor suppressors contribute to the final malignant phenotype (72). The *Eµ-Hnrnpk* mouse model is not an exception to these problems. The limitations of the model are in fact consistent with other B-cell malignancy models. hnRNP K is also a master regulator of multiple biological process, as it regulates transcription, translation, DNA damage response, and splicing (36), which may contribute to the diversity of phenotypes observed in the *Eµ-Hnrnpk* model. Moreover, hnRNP K, as an RNA-binding protein, governs the expression of other master regulators beyond c-Myc (39). There are also multiple lines of evidence that suggest hnRNP K can function as a master cancer switch, driving not only hematological neoplasms, but also solid tumors (42–45).

One of the most common lymphomagenesis mouse models used is the *Eµ-Myc* mouse model. The extensive use of the *Eµ-Myc* model is not only due to historical reasons (model was developed in 1985), but also due to the biological mechanisms of lymphomagenesis. c-Myc is the most widely studied master regulator of B-cell malignancies and is altered in follicular lymphoma, DLBCL, Burkitt’s lymphoma, and myeloma. However, dysregulation of the c-Myc pathway is observed more frequently than can be explained *via MYC* genomic aberrations. Thus, it is critical to identify c-Myc regulators that contribute to its non-genomic regulation. To this end, hnRNP K as an RNA-binding protein emerged as an idyllic candidate, and the *Eµ-Hnrnpk* mouse model allows exploration of B-cell malignancies in the absence of *MYC* alterations. Moreover, we recently found that hnRNP K is overexpressed in DLBCL patients, suggesting its direct role in B-cell malignancies and thereby making the *Eµ-Hnrnpk* model relevant beyond its function as a genetic and mechanistic tool. However, hnRNP K is a relatively under-studied molecule in cancer, particularly in B-cell neoplasms. Our studies with *Hnrnpk* haploinsufficient mice demonstrated that hnRNP K plays a vital role in lymphomagenesis beyond its role as a c-Myc regulator (40). Rigorous experimentation is required to better determine the role of hnRNP K in lymphocyte biology and its implications in development of DLBCL and other B-cell malignancies. Further work also needs to be done to identify the mechanisms underlying hnRNP K overexpression in human disease.

Perhaps the biggest drawback of most common B-cell malignancy mouse models is the diversity in phenotype development that makes them divergent from human disease. As previously discussed, the nature of the oncogenic drivers and their relationship with other cancer regulators combined with the spatio-temporal expression of the genetic modifications contributes to these divergences. Thus, more selective and specific mouse models, using promoters of one-stage/one-grade of B-cell maturation could help to surpass these limitations. Moreover, the generation of more complex mouse models that could recapitulate human diseases more precisely, with a combination of different genetic alterations, could guarantee more consistent models. In fact, complex models, such as *Eμ-Myc/BCR^HEL^/sHEL* to mimic Burkitt’s lymphoma, or *IL-14α TG* × *c-Myc TG (DTG)* mice for blastoid-variant mantle-cell lymphoma (MCL-BV) are good examples (73, 74).

The ability of a murine model to accurately mimic human disease is more relevant when animal models are used as therapeutic platforms. The selection of a model that does not accurately recapitulate human disease could contribute to the low correlation between results observed in pre-clinical and clinical trials. Indeed, only 8% of pre-clinical animal model research successfully advances to clinical trials, and of the experimental agents that are successful in preclinical models, 85% of them fail in early stages of human trials (75). Hence, a battery of B-cell malignancies drivers and promoters is needed to more precisely mimic the spatio-temporal compartmentalization of alterations to ensure better clinical correlations. To this end, the *Eµ-Hnrnpk* model adds to the catalog of mouse models available to study B-cell malignancies and establish therapeutic platforms, alone or in combination with other drivers.

## Ethics Statement

The animal study was reviewed and approved by Institutional Animal Care and Use Committee at MD Anderson under protocol 0000787-RN02.

## Author Contributions

PM, MV-E, and PA-G: conceptualized, organized, and wrote the manuscript and generated figures. MJLA, LEC, and XZ: critically revised the manuscript. SMP and MG supervised and critically revised the manuscript. All authors contributed to the article and approved the submitted version.

## Funding

This study has been supported a National Cancer Institute/National Institutes of Health Award (R01CA207204) and Leukemia and Lymphoma Society (6577–19) to SMP. PM is a Jane Coffin Childs Memorial Fund for Medical Research Fellow and MA is a recipient of the Dr. John J. Kopchick Fellowship. MG is supported by financial support from the Cris contra el Cancer association and Instituto de Salud Carlos III (Ministry of Economy, Industry and Competitiveness) and cofunded by the European Regional Development Fund with Miguel Servet (CP19/00140) and PI (PI18/00295).

## Conflict of Interest

The authors declare that the research was conducted in the absence of any commercial or financial relationships that could be construed as a potential conflict of interest.

## Abbreviations

DLBCL, diffuse large B-cell lymphoma; hnRNP K, heterogeneous nuclear ribonucleoprotein K; VDJ, variability, diversity, and joining; PDX, patient-derived xenograft; IgH, immunoglobulin heavy; RBP, RNA-binding protein; GC, germinal center; SHM, somatic hypermutation; CSR, class-switch recombination.
